# Acceptability of prison-based take-home naloxone programmes among a cohort of incarcerated men with a history of regular injecting drug use

**DOI:** 10.1186/s12954-018-0255-5

**Published:** 2018-09-21

**Authors:** Michael Curtis, Paul Dietze, Campbell Aitken, Amy Kirwan, Stuart A. Kinner, Tony Butler, Mark Stoové

**Affiliations:** 1 Behaviours and Health Risks, Burnet Institute, 85 Commercial Road, Melbourne, Victoria 3004 Australia; 2 Disease Elimination, Burnet Institute, Melbourne, Australia; 3 School of Public Health and Preventive Medicine, Monash University, Melbourne, Australia; 4 Centre for Adolescent Health, Murdoch Children’s Research Institute, Melbourne, Australia; 5 Melbourne School of Population and Global Health, University of Melbourne, Melbourne, Australia; 6 Kirby Institute, University of New South Wales, Sydney, Australia

**Keywords:** Injecting drug use, Naloxone, Overdose, Prison

## Abstract

**Background:**

Take-home naloxone (THN) programmes are an evidence-based opioid overdose prevention initiative. Elevated opioid overdose risk following prison release means release from custody provides an ideal opportunity for THN initiatives. However, whether Australian prisoners would utilise such programmes is unknown. We examined the acceptability of THN in a cohort of male prisoners with histories of regular injecting drug use (IDU) in Victoria, Australia.

**Methods:**

The sample comprised 380 men from the Prison and Transition Health (PATH) Cohort Study; all of whom reported regular IDU in the 6 months prior to incarceration. We asked four questions regarding THN during the pre-release baseline interview, including whether participants would be willing to participate in prison-based THN. We describe responses to these questions along with relationships between before- and during-incarceration factors and willingness to participate in THN training prior to release from prison.

**Results:**

Most participants (81%) reported willingness to undertake THN training prior to release. Most were willing to resuscitate a friend using THN if they were trained (94%) and to be revived by a trained peer (91%) using THN. More than 10 years since first injection (adjusted odds ratio [AOR] 2.22, 95%CI 1.03–4.77), having witnessed an opioid overdose in the last 5 years (AOR 2.53, 95%CI 1.32–4.82), having ever received alcohol or other drug treatment in prison (AOR 2.41, 95%CI 1.14–5.07) and injecting drugs during the current prison sentence (AOR 4.45, 95%CI 1.73–11.43) were significantly associated with increased odds of willingness to participate in a prison THN programme. Not specifying whether they had injected during their prison sentence (AOR 0.37, 95%CI 0.18–0.77) was associated with decreased odds of willingness to participate in a prison THN training.

**Conclusion:**

Our findings suggest that male prisoners in Victoria with a history of regular IDU are overwhelmingly willing to participate in THN training prior to release. Factors associated with willingness to participate in prison THN programmes offer insights to help support the implementation and uptake of THN programmes to reduce opioid-overdose deaths in the post-release period.

## Background

People who inject drugs (PWID), including opioids, are disproportionately represented among the approximately 41,200 people incarcerated at any one time in Australian prisons [1–3]. It is estimated that almost half of prison entrants have ever injected drugs [4] and that approximately one quarter have injected in the month prior to incarceration [5, 6]. People released from prison have significantly higher rates of mortality than the general population [7–10], with opioid overdose responsible for a considerable proportion of excess risk, both in Australia [11–16] and internationally [12, 13, 17–19]. Fatal heroin overdoses (heroin only or poly-substance including heroin) have increased every year since 2009 in Victoria, Australia, with 220 overdose deaths involving heroin occurring in 2017 [20]. Among prisoners and people recently released from prison, witnessing and/or experiencing opioid overdose is common [21, 22].

While strategies such as access to prison opioid substitution therapy have demonstrated some efficacy in reducing post-prison release opioid overdose risk [10, 23], there is considerable evidence that naloxone, an opioid antagonist, delivered via community-based or prison-based take-home naloxone (THN) programmes is effective in reducing the risk of opioid overdose. THN programmes typically train participants in the recognition and management of opioid overdoses and equip participants with naloxone at completion of the training. THN programmes have been found to increase opioid overdose knowledge and reduce opioid-related deaths among those trained and their broader community [24–27]. People who have previously been incarcerated and those involved with community-based correction services report willingness to be trained in how to use naloxone and to use it during opioid overdoses [21, 28–30]. Despite this, published evaluations of prison-based THN programmes and research into the acceptability of THN programmes delivered to people in prison are scarce [31].

Small-scale studies have evaluated the effectiveness of prison-based THN programmes, demonstrating significant improvements in participants’ overdose management knowledge [32, 33], self-reported confidence to manage an opioid overdose [22] and effective actions in overdose simulations [34]. However, very few studies have measured the impact of prison-based THN programmes. A THN programme implemented at the Rikers Island gaol system in New York targeted training and naloxone provision to visitors of inmates identified as being at risk of overdose following release. Of the 283 participants, 40 participants responded to 70 overdose incidents. Among those who witnessed an overdose event, 70% administered naloxone. The survival rate was 94% for the 65 overdose events where data was available [35]. Evaluation of Scotland’s National Naloxone Program reported 11,898 naloxone kits distributed between 2011 and 2013, of which 2273 were given to people leaving prison. Subsequently, a 36% decrease in overdose-related deaths among people released from prison in the previous 4 weeks was observed [36]. Prison-based THN programmes are being established in jurisdictions in the USA [37–39] and Canada. None of these programmes has yet been evaluated.

Very few prisoners have been trained in naloxone provision in Australia. As a component of a broader community-based THN programme, 18 prisoners were trained in overdose prevention and naloxone provision at the Alexander Maconochie Centre in the Australian Capital Territory between 2011 and 2014, while Australia’s first formal prison-based THN programme began at Acacia Prison in Western Australia in 2016 [27, 40]. There is no publicly available data pertaining to the numbers of trainees from the Acacia Prison THN programme. Given the small numbers of programme participants and lack of programme evaluation at present, research is needed to understand who would benefit from participating in a prison-based THN programme, the possible impact of prison-based THN programmes in Australia on post-release opioid overdose, broader opioid overdose reductions among ex-prisoners’ family and social networks and how best to support the implementation and scale-up of prison-based THN. In this article, we examine the acceptability of prison-based THN among a cohort of men who were regularly injecting drugs immediately prior to incarceration and were about to be released from prison^1^ in Victoria, Australia.

## Methods

### Study design

Our data are taken from baseline interviews with 400 participants of the Prison and Transition Health (PATH) Cohort Study. PATH is a longitudinal cohort study of 400 men recruited in the weeks prior to release (baseline interview) from one of the three prisons in Victoria, Australia, who will be followed for 24 months after release. Operational limitations within Victoria’s female prisons at the time of data collection prevented the recruitment of women into PATH. Eligibility criteria included reporting regular injecting drug use (IDU) in the 6 months prior to incarceration (defined as at least monthly IDU), being aged over 18 years, providing informed consent and expecting to be released within 12 weeks of the baseline interview. Participants were recruited from one minimum (*n* = 108), medium (*n* = 111) and maximum (*n* = 181) security facility. The maximum security site was over-sampled to reflect its increased prison population and annual discharges. Recruitment methods included the use of posters, presentations at alcohol and other drug (AOD) group sessions and ‘town hall’ meetings, direct engagement with prisoners during dosing clinics for opioid substitution therapy, workplaces and on prison cell blocks. Prospective participants completed an expression of interest form, which was returned directly to researchers, to AOD programme staff or in secure mail boxes around the prisons. Upon receiving an expression of interest form, researchers provided the prospective participant with information about the study while screening for eligibility. Baseline interviews were conducted between September 2014 and May 2016. Participants completed a researcher-administered structured questionnaire that included socio-demographics, physical and mental health, substance use, criminal activity and pre-release support planning. The mean duration of baseline interviews was 45 min (SD, 12 min; range, 26–73 min) and interviews were conducted a median of 33 days (IQR, 13–62 days) prior to release. Ethics approval for the PATH study was obtained from the Alfred Hospital Human Research Ethics Committee (79/12) and the Victorian Department of Justice Human Research Ethics Committee (CF/14/10169).

### Outcome measures

Participants’ beliefs and attitudes towards the acceptability of THN programmes were assessed through four questions in the baseline questionnaire: (1)'Would you be willing to be trained on how to use naloxone in prison and receive some to take with you when you are released from prison?’; (2) ‘Would you be willing to be trained on how to use naloxone soon after you are released from prison and receive some to take with you?’; (3)‘If a friend had naloxone and was trained to use it, and you overdosed on heroin, would you want them to use it on you?’; and (4) ‘If you had naloxone and were trained to use it and a friend overdosed on heroin, would you use it on them?’. If a participant did not know what naloxone (or ‘Narcan’, as it was known to many prisoners) was, researchers provided a short description of it and its use. All THN measures had yes/no responses.

### Potential correlates

Potential correlates of willingness to engage in prison-based THN training were selected from a review of literature or were considered factors that could be used to inform a prison-based THN trial. Examined variables included age (< 35 years/35+ years), education (did not complete high school/completed high school or alternative qualifications such as a trade certificate), identifying as being Aboriginal and/or Torres Strait Islander^2^ (yes/no), residential location before prison (metropolitan/regional/other^3^), years since first injected (< 10 years/10+ years), ever overdosed on opioids (yes/no), witnessed an opioid overdose in the past 5 years (yes/no), heroin use in the month prior to incarceration (yes/no), illicit benzodiazepine use in the month before prison (yes/no), daily alcohol use in the month before prison (yes/no), length of sentence (< 204 days/204+ days), number of previous incarcerations (< 5 incarcerations/5+ incarcerations), having ever completed AOD treatment in the community (yes/no), having ever completed AOD treatment in prison (yes/no), having completed AOD treatment in prison during that sentence (yes/no), self-reporting being worried about substance use upon release (not worried/a little worried/very worried) and having injected any drug during the current prison sentence (yes/no/declined to answer).

### Statistical analysis

Frequencies of responses to all questions about acceptability of prison-based THN programmes among PATH participants were generated. Potential correlates were described according to whether a participant reported they would engage in prison-based THN training, and we used bivariate logistic regression to examine associations between correlates and THN response to question 1, ‘Would you be willing to be trained on how to use naloxone in prison and receive some to take with you when you are released from prison?’ A multivariable model was constructed using all variables statistically significant in bivariate analysis. Given the exploratory nature of the research and the number of potential correlates included, a stringent approach for constructing a multivariable model was utilised to minimise overfitting. Only variables which were statistically significant at *p* < 0.05 in bivariate analysis were included for multivariable analysis. A complete case approach was used, resulting in the exclusion of 23 participants from analysis. All analyses were conducted using Stata 14 for Windows [41].

## Results

The median age of participants was 35 years (IQR 30–42 years). Most participants (89%) were born in Australia and 97% reported English as their primary language. Among those born overseas, the median number of years living in Australia was 28 (range 2–51 years). Seventeen per cent identified as Aboriginal and/or Torres Strait Islander. Most (82%) participants did not complete high school or a high school equivalent such as a trade certificate. Half (50%) reported receiving a Centrelink benefit (unemployment/study allowance or a pension) as their primary source of income and approximately one quarter (28%) reported that their accommodation was unstable in the month prior to incarceration. Participants had served a median of five adult custodial sentences (IQR 3–9 sentences) prior to their recruitment sentence. Most (89%) had used heroin at some point in their lifetime. The most commonly reported substances used in the month before incarceration were crystal methamphetamine (84%), heroin (56%) and illicit benzodiazepines (31%). Diverted opioid substitution medications (methadone, buprenorphine or buprenorphine with naloxone) (16%), oxycodone (15%) and morphine (10%) were the most commonly used illicit pharmaceutical opioids in the month before incarceration. Almost half (42%) reported using both heroin and crystal methamphetamine in the month prior to incarceration. Among the 220 participants who had suffered a drug overdose (58%), the median number of overdoses was 3 (IQR 2–5). More than half (62%) reported they had witnessed a drug overdose.

Participants’ responses to THN acceptability questions are shown in Table 1. The majority of participants (81%) reported they would participate in THN training if offered during their sentence, would participate in THN training if offered after release from prison (79%), were willing to use naloxone to revive a friend in the event of an overdose (94%) and were willing to have a friend revive them in the event of an overdose (91%).

**Table 1:** Acceptability of take-home naloxone among incarcerated men who reported regular (at least monthly) injecting drug use immediately prior to incarceration (*n* = 377)

Naloxone question	No (%)	Yes (%)
Would you be willing to be trained on how to use naloxone in prison and receive some to take with you when you are released from prison?	73 (19)	304 (81)
Would you be willing to be trained on how to use naloxone soon after you are released from prison and receive some to take with you?	81 (21)	296 (79)
If a friend had naloxone and was trained to use it, and you overdosed on heroin, would you want them to use it on you?	35 (9)	342 (91)
If you had naloxone and were trained to use it and a friend overdosed on heroin, would you use it on them?	23 (6)	354 (94)

Associations between potential correlates and THN question 1 ‘Would you be willing to be trained on how to use naloxone in prison and receive some to take with you when you are released from prison?’ are described in Table 2. Multivariable analysis showed reporting 10 years or more since first injecting drugs (adjusted odds ratio [AOR] 2.22, 95%CI 1.03–4.77), having witnessed an opioid overdose in the past 5 years (AOR 2.53, 95%CI 1.32–4.82), having ever completed AOD treatment in prison (AOR 2.41, 95%CI 1.14–5.07) and having injected a substance during their current sentence (AOR 4.45, 95%CI 1.73–11.43) were associated with willingness to participate in prison-based THN. Declining to answer whether they had injected a substance during their current period of incarceration (AOR 0.37, 95%CI 0.18–0.77) was associated with being less willing to participate in prison-based THN.

**Table 2:** Participant characteristics and modified logistic regression associations with willingness to engage in prison-based take-home naloxone training among incarcerated men who reported regular injecting drug use immediately prior to incarceration

Variable	No. (%) (*n* = 377)	Yes to P-THN^1^ (%)	*p* > |*z*|	OR (95%CI)	AOR (95%CI)*
Demographics					
Age					
< 35 years old	177 (47)	138 (78)		1	–
35+ years old	200 (53)	166 (83)	0.218	1.38 (0.83–2.30)	–
Age of first IDU					
< 18 years old	214 (57)	180 (84)		1	–
18+ years old	163 (43)	124 (76)	0.052	0.60 (0.36–1.00)	–
Education					
Did not complete high school	309 (82)	246 (80)		1	–
Completed high school^2^	68 (18)	58 (85)	0.285	1.49 (0.72–3.07)	–
Aboriginal or Torres Strait Islander					
No	314 (83)	251 (80)		1	–
Yes	63 (17)	53 (84)	0.444	1.33 (0.64–2.76)	–
Accommodation before prison					
Metropolitan	206 (55)	177 (86)		1	1
Regional	161 (43)	120 (75)	0.006	0.48 (0.28–0.81)	0.57 (0.30–1.08)
Other^3^	10 (3)	7 (70)	0.181	0.38 (0.09–1.56)	0.46 (0.09–2.25)
Drug and alcohol					
Years since first injection					
< 10 years	78 (21)	55 (71)		1	1
10+ years	299 (79)	249 (83)	0.012	2.08 (1.17–3.70)	2.22 (1.03–4.77)
Ever overdosed on opioids					
No	157 (42)	114 (73)		1	1
Yes	220 (58)	190 (86)	0.001	2.39 (1.42–4.02)	1.35 (0.72–2.54)
Witnessed an opioid overdose in last 5 years					
No	142 (38)	101 (71)		1	1
Yes	235 (62)	203 (86)	0	2.58 (1.53–4.33)	2.53 (1.32–4.82)
Used heroin in the month before prison (any route)					
No	165 (44)	124 (75)		1	1
Yes	212 (56)	180 (85)	0.018	1.86 (1.11–3.12)	0.95 (0.49–1.83)
Used illicit benzodiazepines in the month before prison					
No	262 (70)	206 (79)		1	–
Yes	115 (31)	98 (85)	0.138	1.57 (0.87–2.84)	–
Daily alcohol use in the month before prison (28+ days)					
No	300 (80)	237 (79)		1	–
Yes	77 (20)	67 (87)	0.116	1.78 (0.87–3.66)	–
Prison					
Length of sentence					
< 204 days	186 (50)	137 (74)		1	1
204+ days	186 (50)	163 (88)	0.001	2.53 (1.47–4.37)	1.27 (0.65–2.48)
Number of previous incarcerations					
< 5 incarcerations	164 (44)	118 (72)		1	1
5+ incarcerations	213 (56)	186 (87)	0	2.69 (1.58–4.55)	1.83 (0.96–3.46)
AOD treatment in the community—ever					
No	118 (31)	90 (76)		1	–
Yes	259 (69)	214 (83)	0.149	1.48 (0.87–2.52)	–
AOD Treatment in prison—ever					
No	92 (24)	58 (63)		1	1
Yes	285 (76)	246 (86)	0	3.70 (2.15–6.36)	2.41 (1.14–5.07)
AOD treatment in prison—this sentence					
No	197 (52)	150 (76)		1	1
Yes	180 (48)	154 (86)	0.022	1.86 (1.09–3.15)	1.15 (0.54–2.48)
Worried about substance use upon release from prison					
Not worried	130 (34)	99 (76)		1	1
A little worried	150 (40)	120 (80)	0.437	1.25 (0.71–2.21)	0.76 (0.38–1.50)
Very worried	97 (26)	85 (88)	0.032	2.22 (1.07–4.59)	1.55 (0.66–3.67)
Injected drugs in prison during this sentence					
No	191 (51)	147 (77)		1	1
Yes	126 (33)	119 (94)	0	5.09 (2.21–11.71)	4.45 (1.73–11.43)
Declined to answer	60 (16)	38 (63)	0.038	0.52 (0.28–0.96)	0.37 (0.18–0.77)

*Only variables which were significant at *p* < 0.05 during bivariate analysis were included in multivariable analysis

^1^P-THN refers to whether the participant reported willingness to participate in prison-based THN training as asked in Q1

^2^Includes high school completion equivalent (e.g. technical, further or industry-specific education courses)

^3^ Other denotes people who were itinerant in the month prior to incarceration and were unable to provide a postcode

## Discussion

Prison and community-based THN training was endorsed by the large majority of our sample of incarcerated men with recent pre-incarceration histories of regular IDU. Willingness to use naloxone on a friend and willingness to be revived by a friend using naloxone were also extremely high. Our findings provide strong evidence that most Victorian prisoners with histories of IDU will accept THN training and provision of naloxone upon release. In addition, our identification of correlates of willingness to participate in training, such as longer histories of IDU and exposure to AOD treatment in prison, provide useful information for targeting the promotion and delivery of prison-based THN programmes. The reported proportion of people with criminal justice system involvement who report willingness to engage in naloxone training has varied, with between 72% and 90% of study participants reporting willingness to be trained in naloxone administration [21, 29, 42], similar to our findings. However, a study of individuals under community corrections orders in the USA reported variations in the proportion willing to receive opioid overdose training (including naloxone) according to individuals that reported never using opioids (32%) relative to those that reported lifetime use of opioids and had experienced an overdose (72%) and those who had used opioids but never overdosed (59%) [30]. While our unadjusted analysis also found that those who used heroin in the month prior to incarceration and those who had experienced an opioid overdose were more willing to participate in prison-based THN training, neither of these factors remained significant in the multivariable analysis. The higher THN acceptability in our study is likely attributable to participants’ patterns of substance use and exposure to opioid overdoses. As noted, PATH participants reported substantial poly-substance use at baseline interview with most (90%) having ever used heroin, more than half (58%) having experienced an opioid overdose and many (62%) witnessing an opioid overdose in the preceding 5 years. This contrasts with Cropsey et al.’s sample, where half (54%) reported never having used an opioid and few (4%) reported ever witnessing an opioid overdose.

Previous studies of prisoners and of people recently released from prison have found 88–90% of participants reported being willing to administer naloxone to a peer/friend in the event of an overdose [29, 32], consistent with our findings. These high rates of willingness to administer naloxone to peers in the event of an opioid overdose were demonstrated during the UK’s multicentre prison-based naloxone on release pilot randomised control trial (N-ALIVE). N-ALIVE sought to establish the impact of prison-based THN programmes on opioid overdoses among prisoners following release. However, whilst the study was established with the intention of facilitating the use of THN by peers and family to reverse prisoner overdose, two thirds of recorded naloxone administrations from N-ALIVE kits were administered by the prisoner to a peer [43]. We found no previous research explicitly assessing factors influencing THN acceptability among people who were incarcerated at the time of data collection, but the association between witnessing an overdose and willingness to engage in THN training is common among PWID who have been incarcerated [21, 29, 32]. We did not find that prison-based THN acceptability was significantly affected by the types of drugs used in the month prior to prison, in contrast to Cropsey et al. [30], whose study of people on community corrections orders reported significant differences based on whether someone had used opioids. As noted above, these differences may be due to differing patterns of substance use and previous exposure to opioid overdose between samples.

The individual factors associated with high acceptability of prison-based THN reported in our study provide insights to support its implementation and uptake in Victorian prisons and elsewhere.

Assessments of prisoner needs on reception at prison or during sentences could be used to identify people who may use opioids upon release and be at risk of opioid overdose, those who have witnessed opioid overdoses and those who had injected drugs over longer periods for targeted promotion of prison-based THN training. A systematic review of peer-based prison intervention programmes found that peer-based programmes can be effective in improving blood-borne virus (BBV) knowledge, reducing in-prison BBV transmission risk behaviours and improving mental health [44]. Individuals with lived experiences related to long-term IDU and witnessing overdose (peers) could also be used to increase uptake and coverage of prison THN programmes or deliver THN training themselves. Referrals to AOD treatment could also include an automatic offer of prison-based THN training, or training could be integrated into programmes that include AOD treatment provision.

Our finding that heroin use in the month before incarceration was not significantly associated with THN acceptability supports making prison-based THN programmes broadly accessible to all prisoners, rather than targeting THN programmes towards people based on types of substance use pre-incarceration. While in the context of community corrections others have found that those with histories of opioid use were more likely to report willingness to engage in THN training [30], the outcomes reported from the Rikers Island THN programme, in which almost half of the naloxone administration events from participants occurred with strangers [35], and the N-ALIVE trial, in which two thirds of naloxone administration events were administered by the prisoner to another person [42, 43], suggest a broad community-level overdose prevention benefit from a prison THN programme. This would require an approach that targets communities (e.g. those impacted most by incarceration) as opposed to individuals for THN programmes. Future research should explore the potential impact of prison-based THN programmes on not only the programme participants but also on their broader networks and communities.

Because people who inject drugs whilst incarcerated are more likely to return to IDU upon release [45], this group should also be targeted for prison-based THN programmes. The finding that people who declined to answer whether they had injected drugs whilst incarcerated were less likely to report willingness to engage in prison-based THN programme highlights the sensitive nature of a prisoner’s IDU status whilst incarcerated. The potential perception among people in prison that THN programme participation equates to an increased risk of being identified as a drug user and being targeted for drug interdiction activities needs to be considered in implementing prison THN programmes.

Our study has several limitations. The accuracy of self-report studies may be influenced by social desirability and recall biases; however, numerous studies have established that PWID can provide accurate recollection of their AOD use histories [46–49]. While these findings are taken from the baseline interviews of a longitudinal study, they are in essence cross-sectional; as such, causation cannot be established. Prospective data collection will allow for identification of individuals who overdose, allowing for retrospective identification of pre-release factors associated with increased overdose risk. This will allow improved targeting of harm reduction interventions, including prison-based THN programmes, to help reduce post-release overdose risk. As PATH recruitment criteria precluded women, youth and remanded prisoners, our findings may not be generalisable to these groups. Our decision to only include potential correlates in the multivariable model which were significant at *p* < 0.05 in bivariate analyses may have resulted in the omission of additional potential correlates. Differences between poly-drug types typically associated with opioid overdose in Australia (typically benzodiazepines and/or opioid pharmacotherapies, in combination with heroin) [20] and other countries (e.g. overdoses driven by synthetic opioids such as fentanyl in the USA or Canada [50]) may limit the utility of our findings for international prison-based THN programmes. Given the overlap of non-injecting opioid use in prison with IDU in prison categories, we were unable to assess the independent relationship between willingness to participate in a prison THN programme and non-injecting opioid use. As we were unable to collect data from people not participating in PATH, we are unable to comment on the acceptability of prison-based THN among non-participants. Finally, our findings do not explore how the content, location, length or training provider of a prison-based THN programme may influence willingness to engage training. Future explorations of prison-based THN acceptability should broaden to explore these factors.

## Conclusion

People exiting prison face increased risk of opioid overdose in the months following release. With naloxone available, opioid overdoses are rarely fatal. The overwhelming level of interest among male prisoners with a history of IDU in our study about participating in prison-based THN programme training underscores the crucial role of prison-based THN programmes in preventing mortality among opioid users. This finding, alongside the range of correlates of willingness to participate in prison-based THN programme training, provides insights to help maximise prison-based THN programme uptake and its impact on overdose mortality prevention in Australia and elsewhere.

## Abbreviations

AOD: Alcohol and other drugs
AOR: Adjusted odds ratio
IDU: Injecting drug use
NFA: No fixed address
OR: Odds ratio
PATH: Prison and Transition Health Cohort Study
PWID: People who inject drugs
THN: Take-home naloxone

## Footnotes

1 Australian prisons are operated by state governments. Victoria’s prison system consists of 15 facilities—13 for men and 2 for women. Victorian facilities house minimum, medium or maximum security prisoners exclusively. There are no differences between a correctional centre and prison, and prisoners frequently move between facilities. Prisoners are assigned security ratings based on factors including the nature of their offence, length of sentence, previous offences and incidents whilst incarcerated. Prisoners are detained at a facility in accordance with their security rating. Security ratings are adjusted based on prisoner behaviour.

2 In Australia, Aboriginal and/or Torres Strait Islanders are typically grouped in population research to encompass all Indigenous Australians, whilst recognising the distinct cultural heritage of each group. People identifying as Aboriginal and/or Torres Strait Islanders are over-represented among people in prison and people reporting IDU and are therefore routinely distinguished in related research.

3 ‘Other’ includes people who were itinerant in the month before incarceration and were unable to provide a postcode of residence in the month before prison. Metropolitan and regional residential locations also include people who were homeless but able to provide residential postcodes.
